# Social Network, Food Patterns, Physical Activity and Associations with Overweight and Obesity in Adolescents from a School in Rural Brazil

**DOI:** 10.3390/nu15153305

**Published:** 2023-07-26

**Authors:** Maria de Jesus Xavier Aguirre, Flavia Cristina Drumond Andrade, Moisés Alberto Calle Aguirre, Josivan Ribeiro Justino, Bruna Leal Lima Maciel

**Affiliations:** 1 Graduate Program in Health Sciences, Federal University of Rio Grande do Norte, Natal 59078-970, Brazil; 2 School of Social Work, University of Illinois at Urbana-Champaign, Urbana, IL 61801, USA; 3 Graduate Program in Demography, Federal University of Rio Grande do Norte, Natal 59078-970, Brazil; 4 Computer Science Academic Department, Federal University of Rondônia, Porto Velho 76808-695, Brazil; 5 Department of Nutrition, Federal University of Rio Grande do Norte, Natal 59078-970, Brazil

**Keywords:** obesity, overweight, food patterns, adolescents

## Abstract

The objective of this study was to evaluate the social network, food patterns, physical activity, and their associations with overweight/obesity in adolescents from a school in rural Brazil. Students from a rural school in Northeast Brazil (*n* = 90) completed questionnaires on sociodemographic characteristics, food consumption, physical activity, and a name generator. Social networks were constructed using students’ social proximity ties. Principal component analysis was performed to determine food patterns, and logistic models were used to investigate variables associated with overweight/obesity. Most participants were girls (62.9%), and the proportion of overweight/obesity was 30% among adolescents. Students cited 2070 people from their networks (family, friends at school, friends outside of school, and others). Among them, the family had the highest degree of influence (61%) in the network and had the most shared meals with adolescents (47%). Adolescents’ perception of their family members’ body size as obese, compared to normal or underweight, was prevalent (51%). Adolescents with unhealthy food patterns were 72% more likely to be categorized as overweight/obese, and eigenvector centrality was also associated with overweight/obesity (OR = 5.88, 95% CI = 1.08–32.03). Adolescents presented a social network with strong family influence, in which a high percentage of overweight/obesity was observed. Adolescents with high eigenvector centrality were more likely to be in the overweight/obesity category. Additionally, overweight/obesity was associated with unhealthy food patterns in the family network.

## 1. Introduction

Obesity has been a global concern despite joint efforts by nations to set goals to slow its rise. In the last three decades, no countries have had clear downward trends in obesity [1,2]. In Brazil, overweight and obesity in the school-age population continue to grow in all regions. The prevalence of overweight among adolescents aged 15 to 17 years was 19.4%, corresponding to 1.8 million people, and higher among female adolescents (22.9%) than male adolescents (16.0%). Regarding obesity, the prevalence among female adolescents (8.0%) was also higher than among male adolescents (5.4%) [3]. The prevalence of obesity in Brazil is smaller in rural areas than in urban areas [4]. Nevertheless, it is growing, following overall obesity increases in recent decades [4], and studies related to lifestyle and eating habits are still limited in rural areas [5].

Unhealthy eating habits and low physical activity are social behaviors shared among family, friends, schoolmates, and neighbors; that is, the behavioral norms of friendship groups can spread through the social network, influencing or not the development of overweight or obesity [6,7,8]. This type of approach in studies that focus on the role of social networks on the incidence of obesity is still little studied in Brazil. 

Social networks refer to a set of individuals and the ties between them, constituting a comprehensive term that may include a type of social relationship, social bond, social norm, and social influence [9]. However, the individual’s social network behaves as a dynamic structure that conditions their behavior in various aspects of life; these relationships circulate through the channels of the network that determine the existence or absence of some relationship between individuals [9]. The network implies a tissue of relationships through which information, help, and advice are transferred [10].

These two findings—the absence or scarcity of the use of social network analyses (SNA) in obesity investigations in Brazil and its increasing incidence or prevalence in rural areas—constitute the guiding concern of this study.

Obesity is linked to several complications, such as metabolic, endocrine, cardiac, respiratory, renal, immunological, and psychological changes, which can impact health in the short and long term [11,12]. In adolescence, the long-term effects of obesity may also include cancer and diabetes and greater difficulty in changing inappropriate eating habits [12], which may lead to related cardiometabolic complications in adulthood [13]. Furthermore, obesity in childhood and adolescence predisposes to greater chances of obesity in adulthood [14].

The main factors for this trend are related to lifestyle, the leading environmental cause of overweight in adolescents, especially the high consumption of high-fat, high-calorie foods, reduced physical activity, and a sedentary lifestyle [15].

In this sense, social or friendship networks can lead to social contagion processes. Once incorporated into this network, the person can be influenced by those who have a more central position [16] within the network and acquire weight over time [7,16], incorporating obesogenic habits [17,18], such as eating large amounts of highly processed and fast food. The opposite situation or behavior is expected from individuals associated with social networks or friendships composed of individuals with healthier eating and behavioral patterns; that is, they are more likely to incorporate non-obesogenic habits.

The first researchers who studied the concept of social contagion through social networks were Christakis and Fowler [8], who started the debate on the spread of obesity. After that, authors such as Cohen–Cole and Fletcher [19] added new contributions to the effects of social networks on the incidence of obesity. Other studies that discuss the relationship between obesity and social network analysis also reveal the impact of the social environment on the increase in obesity in the adolescent population [20,21,22].

The objective of this study was to evaluate the social network, food patterns, and physical activity and their associations with overweight/obesity in adolescents from a rural school in Brazil. The study hypothesis is that social networks, food patterns, and physical activity are associated with overweight/obesity in adolescents at a school in rural Brazil.

## 2. Materials and Methods

This is a cross-sectional study with data collected from March to December 2019. The sample included ninety high school students from a rural school in Ielmo Marinho, Rio Grande do Norte, Northeast Brazil. The municipality is part of the Metropolitan Region, is fifty-five miles from the state capital, Natal, and its population represents 87.3% of the rural area, according to data from the last census [23].

Participants answered a questionnaire related to sociodemographic characteristics, food consumption, physical activity, and participation in social networks. Participants were randomly selected, with proportional allocation of strata, with a sampling error of 5% and a confidence level of 95%.

The inclusion criteria for the study were being enrolled in the school, being between 14 and 19 years old, not having any metabolic diseases, and, in the case of women, not being pregnant. The research data were collected in a school classroom. Students in the process of being transferred or withdrawn from school activities due to a medical certificate at the time of data collection were excluded from the study.

The Research Ethics Committee approved the study of the Federal University of Rio Grande do Norte (CAAE #2.096. 68318117.0.0000.5292), number: (2.096.000). 

All participants were informed about the research objectives and agreed to participate, signing the informed Consent Term. Minors were included in the study after written informed consent of the mother or legal guardian. Participants did not receive any incentive or reward for participating in the study.

### 2.1. Study Variables

The self-administered questionnaire was based on the National Health School Health Survey (PeNSE) [24], adapted by the researchers, containing questions about sociodemographic characteristics (sex, race, income, and mother’s education); food consumption habits (twelve food groups were researched, but only the first seven were considered for this study—(i) fresh fruit or fruit salad, (ii) vegetables, (iii) sweets, (iv) fried snacks, (v) soda, (vi) salty industrialized/ultra-processed foods, (vii) stuffed biscuits, (viii) beans, (ix) rice, (x) red meat, (xi) white meat and milk and derivatives and the routine of how many days a week the participant had breakfast); and physical activity (days the participant had physical education classes at school, the number of hours the student spent sitting during the day, screen time for playing video games, time using the computer, talking with friends on the cell phone and the number of hours a day spent watching television). Adolescents were also interviewed and answered a questionnaire about social networking questions.

### 2.2. Anthropometric Measurements

Trained nutritionists took participants’ height and weight measurements in a designated room to ensure privacy. Following the researchers’ recommendation, participants wore light clothing without shoes or socks.

Weight was measured on a digital anthropometric scale (Tanita brand, model Bc 1500), with a resolution of 100 g and a capacity of 150 kg. Height was measured using a mobile stadiometer. The body mass index (BMI) was calculated as the ratio between weight (kg) and height squared in meters and classified according to criteria established by the World Health Organization (WHO), according to sex and age [25]. The z-scores for each student were obtained using the growth curves proposed by the WHO [25]. Adolescents were classified according to the BMI-for-age cut-off points as “normal weight” for z scores ≥ −2 and ≤+1; “overweight” for z scores > +1 and ≤+2; “obese” for z scores > +2 [25]. The weight variable was dichotomized into normal weight as a reference and overweight/obesity, resulting from the combination of overweight and obesity.

### 2.3. Social Networks

The research on social networks was performed through a questionnaire, where the student was asked to name people in their relationship and provide information about the quality of the relationship. A trained interviewer registered the responses [26,27].

Participants were asked to name up to 23 important people who were part of the group of their relationships to measure the close ties of students. Subsequently, the interviewees recorded the sex of each person cited, age, perception of the type of body of the cited person (normal, underweight, and overweight/obese), the degree of relationship with that person (family, friends from the school, and neighborhood), the relationship of influence (weak, medium and strong) and whether they ate in the company of that person (yes/no). Each student interviewed corresponds to an “ego”, and the 23 names mentioned by the interviewee correspond to the “alters” of their relational environment. The ego and the alters are social actors in the studied network [26,28].

### 2.4. Food Patterns and Breakfast Consumption

Food consumption indicators were obtained from the question: In the last 7 days, how many days did you consume “food group”? (1 day in the last 7 days, 2–6 days, and every day in the last 7 days). The student recorded the number of days he/she consumed the following food groups of the seven food groups considered: (i) fresh fruit or fruit salad; (ii) vegetables; (iii) sweets (candies, chocolates, chewing gum, or lollipops); (iv) fried snacks (such as chicken drumstick, fried kibbeh, or French fries); (v) soda; (vi) salty industrialized/ultra-processed foods (such as hamburgers, ham, bologna, salami, sausage, instant noodles, packaged snacks or salted crackers) and (vii) stuffed biscuits.

This set of variables was used to determine the food pattern by applying the principal component analysis (PCA) technique. This methodology allows the collected food consumption variables to be reduced to a smaller set of variables; that is, it selects the more expressive variables that configure food patterns, preserving as much variability as possible [29,30]. PCA and Factor Analysis are two techniques widely used in multivariate data analysis. Both are statistical methods used to reduce the dimensionality of the data. Factor analysis aims to identify latent relationships between a set of observed variables; that is, it identifies underlying or latent factors that explain most of the variance in the data [30].

The food groups that contributed to the characterization of each food pattern had a factorial load (≥0.30); variables within this value are considered to meet the minimum necessary for the interpretation of the structure, and values below this level were eliminated [31].

The number of components was built based on the scree plot, and it was possible to identify two groups to represent the main component used in the grouping.

For PCA, component 1, which represents the first extracted food pattern (stuffed biscuits, sweets, processed foods, soda, and fried snacks), represented the largest portion of the variability of the set of variables and explained 34% of the variability, and component 2, the second pattern food (vegetables and fruits), contributed 22%, totaling 57% of the explained variability.

Thus, the food patterns obtained based on the PCA were grouped into healthy and unhealthy. For our study, we defined unhealthy food patterns when individuals consumed foods such as stuffed biscuits, sweets, industrialized, soda, and fried snacks and healthy food patterns as those who consumed fruits, fruit salad, and vegetables. In addition, cluster analysis was used using the non-hierarchical k-means method to categorize healthy and unhealthy food patterns.

The question “Do you usually have breakfast? (yes, every day; 5 to 6 days; 3 to 4 days; 1 to 2 days; not and rarely)” was evaluated as a variable reflecting the eating routine. The variable was recategorized into having breakfast regularly (yes, every day; 5 to 6 days; 3 to 4 days; 1 to 2 days) and having it irregularly (not or rarely having breakfast).

### 2.5. Physical Activity and Screen Time

Physical activity practiced at school was also evaluated, with the question: How many days did you have physical education classes at school (0–5 days), dichotomized into (No days a week and 1 to 2 days a week, since none of the students, interviewed reported having more than two physical education classes a week). Exposure to sedentary behaviors was evaluated based on the number of hours the student spent sitting during the day (Monday to Friday), screen time for playing video games, time using the computer, talking with friends on the cell phone (up to 1 h a day, more than 2 h, 3–4 h, 5–7 h, and 8 h a day), and the number of hours a day spent watching television, also on five days of the week except for Saturdays, Sundays and holidays (Do not watch TV, up to 1 h, 2 h to 4 h and more than 4 h), both recategorized in up to 2 h a day and more than 2 h a day.

### 2.6. Confounding Variables

Confounding variables included the following sociodemographic variables: Sex (male/female), race (yellow, white, black, and pardo/brown), income (less than one minimum wage and more than 1 minimum wage), and mother’s education (incomplete elementary school, incomplete elementary school and complete high school/incomplete and complete higher education) grouped into (<9 years of study, 10 to 11 years and 12 years or more of study).

### 2.7. Data Analysis

Descriptive statistics were used to examine sociodemographic, food consumption, and physical activity data. The chi-square test was used to test the association between the analyzed variables. 

The principal component analysis (PCA) method was used to identify food consumption patterns, considering the weekly frequency of healthy and unhealthy food markers. The PCA was followed by varimax rotation. The number of components was constructed based on the scree plot, identifying two groups representing the main component used in the clustering. These groups were classified into healthy and unhealthy dietary patterns through cluster analysis using the non-hierarchical “k-means” method.

For each network of egos, the eigenvector centrality measure was calculated. This metric considers not only the connections of a given actor but also the connections of the actors that connect to it. Thus, it measures the importance of the actor in the network with the significance of his neighbors and is associated with the actor’s prestige and the power of influence he exercises in the network [32].

Logistic models were then used to assess the association between overweight and obesity and sociodemographic variables, dietary patterns, and networks. The association between overweight and obesity and the variables of interest was determined by logistic regression, calculating odds ratios (OR) in each case with the respective 95% confidence intervals (CI). The level of statistical significance (*p*-value) of the values was set at *p* ≤ 0.05.

After selecting the variables, the goodness of fit of the final model was verified using the Hosmer and Lemeshow test and the ROC curve, that is, whether the model was efficient in describing the relationship between the dependent variable (overweight/obesity) and the independent variables (food consumption, physical activity, and social networks). For the test, the generalhoslem package of the R software (version 1.3.4) was used [33].

The result value of the area under the ROC curve found was 0.7 ≤ AUC ≤ 0.8, considered acceptable according to the parameters [34].

Analyses were performed with R Studio software (version 2023.03.0) as the primary tool for statistical analysis and Ucinet (version 6 for Windows) Net Draw for network analysis. 

Post hoc sample calculation (https://clinical.com/stats/Power.aspx (acessed on 28 March 2022)) was performed using the incidence percentage of overweight/obesity of 19.3% [35], with an alpha value of 0.05. In order to evaluate the probability type II error (1—the statistical power), we calculated the statistical power of the study was 70.1%. Power analysis was also conducted a posteriori for the Chi-square test (X2) and the logistic regression, considering the sample size (*n* = 90) and alpha at 0.05, using GPower software (version 3.1.9.7). The achieved power was 70.1%, assuming a large effect size at 0.5 for the X2, and 97%, assuming an odds ratio = 3.0 for the logistic regression.

## 3. Results

Two participants were excluded from the study for presenting incomplete information, and missing values were not recorded in the dependent variable as in the independent variables.

The data presented in Table 1 show that half (51.1%) of respondents were aged 16–17, the mean age of the studied population was 16.5 years, and 64.4% were female.

More than half of adolescents declared to be of mixed race/color (57.8%) and with an income of up to 1 minimum wage (78.9%). Most declared having a healthy dietary pattern (60.0%) and a normal BMI (70.0%). Most mothers of the adolescents interviewed (61.1%) had 9 to 11 years of schooling.

**Figure 1:**
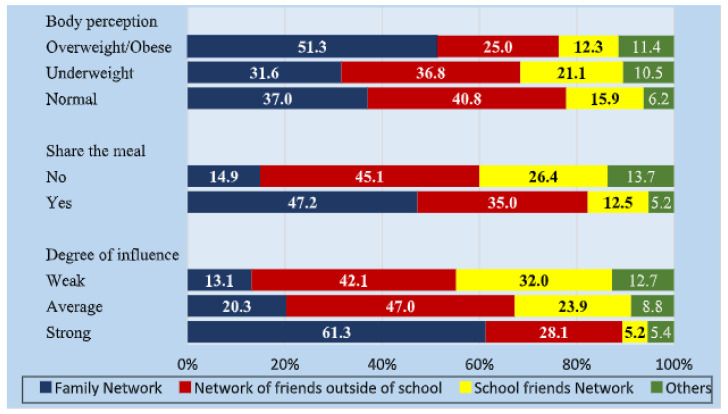
Characteristics of alters, types of networks, degree of influence, and sharing of meals reported by adolescent students from a school in rural Brazil.

The 90 students interviewed named 2070 alters with the various types of networks: family (*n* = 772), school friends (*n* = 789), friends outside school (*n* = 347), and significant others (*n* = 162). People mentioned in the family network had the strongest degree of influence (61%), followed by friends outside of school (28%). People from the family network were referred to as the ones who most shared meals in the company of the interviewees (47%). Regarding the perception of body type, the obese type prevailed in the description of the members of the family network (51%) and was associated with 25% of the members of the network who were friends outside of school. Considering the underweight type, 36.8% belonged to the out-of-school friends’ network, 31.6% to the family network, and 21.1% to the school friends’ network. Among the alters described as having a normal body, members of the friend network outside of school predominated (40.8%), followed by those belonging to the family network (37.0%) and the school friends’ network (15.9%) (Figure 1).

Regarding the people with whom the students had meals, members of the family network (47.2%) predominated, followed by the network of friends outside school (35.0%). Among the alters who indicated a strong degree of influence on the students, there was a large predominance of the family network (61.3%), followed by alters belonging to the friend network outside school (28.1%). All variables evaluated showed a strong association with the type of network variables (*p* < 0.01) (Figure 1).

The binary logistic regression statistical technique was used to analyze the association between food patterns, consumption of breakfast, and physical activity with overweight/obesity. Covariates were selected using the stepwise method that allowed us to arrive at two models. Table 2 registers the independent variables and the statistics generated by logistic regression for Model 1 (unadjusted) and Model 2 (adjusted).

The Hosmer and Lemeshow [33] test, according to the area under the ROC curve, showed practically an equal percentage of correct answers (73%) for both models (Figure S1), a value that means that the two models presented good discrimination power (0.7 ≤ *AUC* ≤ 0.8) [34]. However, the second model was the best as it had the lowest AIC value (110.33) and good AUC discrimination power.

Thus, if two or more models are well adjusted and have adequate predictive capacity, one should prefer the model that involves the smallest number of parameters to be estimated, which explains well the behavior of the response variable [34], and the adjusted model 2 was selected, as it was the most parsimonious model [36].

Table 2 presents the results of the multivariate analysis in which the variables of the adjusted model food patterns (AOR = 3.12 95% CI = 1.17–8.29) and breakfast (AOR = 2.89 95% CI = 1.05–7.92) showed a positive association for the dependent variable overweight/obesity. The odds of being overweight/obese were greater among adolescents who had breakfast irregularly than those who ate breakfast regularly (AOR = 2.89 95% CI = 1.05–7.92). Regarding food patterns, the odds of being overweight/obese were greater among adolescents who consumed unhealthy foods than adolescents who consumed healthy foods (AOR = 3.12 95% CI = 1.17–8.29).

Logistic regression models were used to analyze the association between the dependent variable (overweight/obesity) and each of the representative independent variables (food patterns, sex, and eigenvector centrality). Table 3 registers the explanatory variables and the statistics generated by logistic regression for Model 1 (unadjusted) and Model 2 (adjusted).

In this second analysis, the quality of the fit of the final model was also verified through the Hosmer and Lemeshow test and the ROC Curve (AUC) [34]. In addition, AUC values were used to compare the models. The Hosmer and Lemeshow test, according to the area under the ROC curve, showed practically an equal percentage of correct answers (75%) for both models (Figure S2), a value that means that the two models presented good discrimination power (0. 7 ≤ *AUC* ≤ 0.8) [34]. However, model 2 (adjusted) was the best, having the lowest AIC value (101.43) and good AUC discrimination power.

The chance of being overweight/obese was greater among male students than female (AOR = 4.18, 95% CI = 1.44–12.16). As expected, students with an unhealthy food pattern were more likely to be overweight/obese than those with a healthy one (AOR = 3.74, 95% CI = 1.35–10.38). In the case of the relationship between overweight/obesity and the network variable eigenvector centrality, adolescents who had a network with a higher degree of eigenvector centrality, that is, had central alters of influential intermediation with greater proximity, presented a greater chance of overweight/obesity in relation to adolescent students who had a network with a lower degree of eigenvector centrality (less influential alternates) (AOR = 5.88, 95% CI = 1.08–32.03).

**Figure 2:**
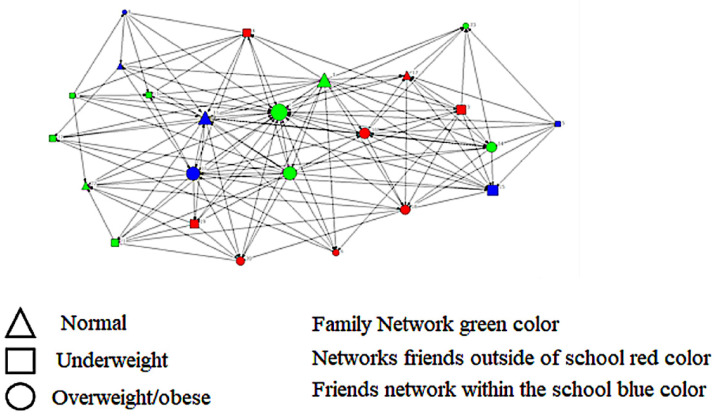
The social network of the adolescent number 40 (with obesity), belonging to the group of students from a school in rural Brazil, due to the centrality of the eigenvector.

This can be illustrated in the individual network of adolescent number 40 in Figure 2, where prestige is indicated by the node’s size (point of connection between the relationships). Adolescents with overweight/obesity (represented by circles) obtained larger sizes in relation to other members of the network who presented normal weight (represented by triangles).

## 4. Discussion

This study demonstrated the association between overweight/obesity, food patterns, and physical activity using the analysis of social networks in adolescent high school students in a rural area of northeast Brazil. We also found that family and friends networks potentially influence food patterns. The influence level depended on the egos’ behaviors and the perceptions that the egos had of the alters. The family represented a high degree of influence within the social networks of the interviewees.

At the same time, some studies [37,38] have already suggested that the family meal environment offers an opportunity to discuss and disseminate values and beliefs, contributing to the formation of relatively homogeneous behavior patterns nuanced by culture and historical context [38].

Some research has shown that students’ networks can influence eating behaviors, physical activity, and obesity [21]. Therefore, it seems essential to understand not only the composition and structure of these networks and identify the socially connected actors but also their influences on health behaviors that can help prevent obesity and lead to interventions related to this problem [38,39].

In the case of this study, adolescents mainly gathered with their families to have their meals, and the family network exerted a more significant influence on food patterns. In addition, adolescents revealed a greater perception of body type, such as obese or overweight, within the family. These results align with those indicating that social ties can influence people’s behavior [40,41]. These ties must be strengthened when they do not disseminate risk behaviors to health and are identified as significant for adopting healthy eating habits and physical behaviors. Conversely, ties could be reduced when they negatively influence food and life habits and disseminate risk behaviors for obesity [42,43].

The influence of the family and the perception of the body as obese within the family network suggest the possibility of adopting intervention strategies aimed at improving the eating behavior of several members of the network, as their influences can last until adulthood.

In our study, the eigenvector centrality indicator was significant. It measures the importance of the actor in the network by the significance of its neighbors. For example, a student with few connections could have a high eigenvector centrality if those few connections were with important connection actors who had attributes of significant influence and prestige with other students. These network interaction channels would be the conduits that are associated with the exchange of ideas and dissemination of information on obesogenic habits that are disseminated in the network structure. These results are similar to those reported by other researchers using SNA [44,45,46].

Due to the characteristic of the influencing force of the indicator in the network, these results may have important implications for the spread [8] of obesity that can start at a young age and, in the long term, have consequences in adulthood [46,47,48].

In our data, the regular habit of having breakfast was a protective factor against weight gain. This finding leads us to reflect on the importance of breakfast, reinforcing that its composition is generally associated with higher consumption of fruits, vegetables, milk, and whole grains [48,49]. This may explain its association with lower chances of overweight/obesity in our study. Unhealthy eating practices, the availability of high-calorie foods, and the easy accessibility of these foods have been recognized in studies as contributing to the growing trend of obesity [50,51,52]. Furthermore, studies show that ultra-processed foods contribute to the diets of adolescents in many countries and represent a nutritional profile consistent with an increased risk of obesity [53,54], corroborating our study, which found that unhealthy food patterns composed of processed foods were related to greater chances of obesity in adolescents.

A limitation of this study is the homogeneity of the sample, as the data collection took place in only one school. Thus, the results found cannot be generalized. Nevertheless, other studies have a similar sample [5,21,43]. Given the small sample size, the wide confidence intervals for some variables limit the precision of our estimates. Such limitations, however, do not invalidate the study because many alters were mentioned in the network (*n* = 2070), allowing significant variability in the social network analysis. Further, the a posteriori power tests analysis indicated reliable results. In addition, relying on adolescent self-report data might have also given bias, which is not circumventable in this kind of study. Nonetheless, this study presents strengths: It examines social networks, food patterns, and physical activity and associations with being overweight/obese in high school students from rural northeast Brazil. Our study not only focuses its analysis on the network of friends inside the school but also covers the family network and the networks of friends outside the school. In addition, it provides relevant information that can contribute to public policies aimed at being overweight/obese, providing a new approach to intervention to this public health problem.

Future studies should consider collecting data on aspects related to the environment surrounding sports facilities and places to buy food close to schools that can influence the behaviors of adolescents concerning their lifestyles associated with being overweight/obese. Studying urban schools to observe possible contrasts will also be important. Finally, implementing interventions related to being overweight/obese that use the methodology of complex-agent-based systems based on the family network could reduce the prevalence of overweight and obesity in this population.

## 5. Conclusions

The research showed that social networks in a sample of adolescents in a rural area in Brazil were mainly defined by family networks and friends inside and outside school, with the most significant network being the family. In addition, the network identified representatives with a high degree of centrality and prestige with the power to disseminate obesogenic habits.

Unhealthy food patterns were also directly associated with overweight/obesity. In the context studied, the results showed that interventions are necessary to reduce overweight/obesity in adolescents, considering both the network structures in disseminating overweight/obesity and food patterns consumption, raising awareness of a healthy diet and lifestyle that adolescents will maintain until adulthood.

## Figures and Tables

**Table 1:** Sociodemographic characterization of adolescent students from a school in rural Brazil 2019.

Variable	*n*	%
**Age** **(in years)**		
14–15	25	27.8
16–17	46	51.1
18–19	19	21.1
Total	90	100.0
**Sex**		
Female	58	64.4
Male	32	35.6
Total	90	100.0
**Race**		
White	23	25.5
Black	15	16.7
Pardo/Brown	52	57.8
Total	90	100.0
**Income**		
≤1 minimum wage	71	78.9
≥2 minimum wages	19	21.1
Total	90	100.0
**Food Patterns**		
Healthy	54	60.0
Unhealthy	36	40.0
Total	90	100.0
**BMI**		
Normal	63	70.0
Overweight/Obesity	27	30.0
Total	90	100.0
**Maternal schooling (in years)**		
<9	10	11.1
9–11	55	61.1
≥12	25	27.8
Total	90	100.0

**Table 2:** Logistic regression results for overweight and obesity among adolescents from a school in rural Brazil, according to food patterns, consumption of breakfast, and physical activity.

		Model 1 (Unadjusted)		Model 2 (Adjusted) *	
Variable	*n*	*p*-Value	OR (95%CI)	*p*-Value	AOR (95%CI)
**Food Patterns**					
Healthy	54	-	-	-	-
Unhealthy	36	0.029	2.99 (1.11–8.03)	0.022	3.12 (1.17–8.29)
**Consumption of breakfast**					
Regular	49	-	-	-	-
Irregular	41	0.039	2.89 (1.05–7.96)	0.039	2.89 (1.05–7.96)
**Hours of tv**					
>2 h	54	-	-	-	-
≤2 h	36	0.529	0.71 (0.24–2.08)	0.474	0.67 (0.23–1.96)
**Screen time/day**					
>2 h	53	-	-	-	-
≤2 h	37	0.996	0.99 (0.35–2.84)	0.847	0.91 (0.33–2.46)
**Physical activity at school**					
No	44	-	-	-	-
Yes	46	0.414	0.65 (0.23–1.83)	0.504	0.70 (0.25–1.95)

* In the adjusted model, race was excluded for a better fit.

**Table 3:** Logistic regression estimates for overweight/obesity according to sex, food patterns, and eigenvector centrality of social networks among adolescents from a school in rural Brazil.

		Model 1 (Unadjusted)		Model 2 (Adjusted)	
Variable	*n*	*p*-Value	OR (95%CI)	*p*-Value	AOR (95%CI)
**Sex**					
Female	58	-	-	-	-
Male	32	0.007	4.44 (1.50–13.17)	0.008	4.18 (1.44–12.17)
**Food patterns**					
Healthy	54	-	-	-	-
Unhealthy	36	0.009	4.08 (1.41–11.76)	0.011	3.74 (1.35–10.38)
**Eingenvector centrality**					
0.00–0.10	16	-	-	-	-
0.11–0.68	74	0.047	5.72 (1.02–32.17)	0.040	5.88 (1.08–32.03)

Note: The adjusted odds ratio (AOR) of the mother’s education and physical education were excluded from the model.

## Data Availability

Data described in the manuscript, code book, and analytic code will be made available upon request pending application and approval.

## Supplementary Materials

The following supporting information can be downloaded at: https://www.mdpi.com/article/10.3390/nu15153305/s1, Figure S1: ROC curve of the adjusted models—model 1 and model 2. Figure S2: ROC curve of the adjusted models—model 1 and model 2.
