# Supplementary material for: Social Network, Food Patterns, Physical Activity and Associations with Overweight and Obesity in Adolescents from a School in Rural Brazil
Source: Nutrients. 2023 Jul 26;15(15):3305. doi: 10.3390/nu15153305 (PMC10421155; doi:10.3390/nu15153305)

Figure S1: ROC curve of the adjusted models - model 1 and model 2.

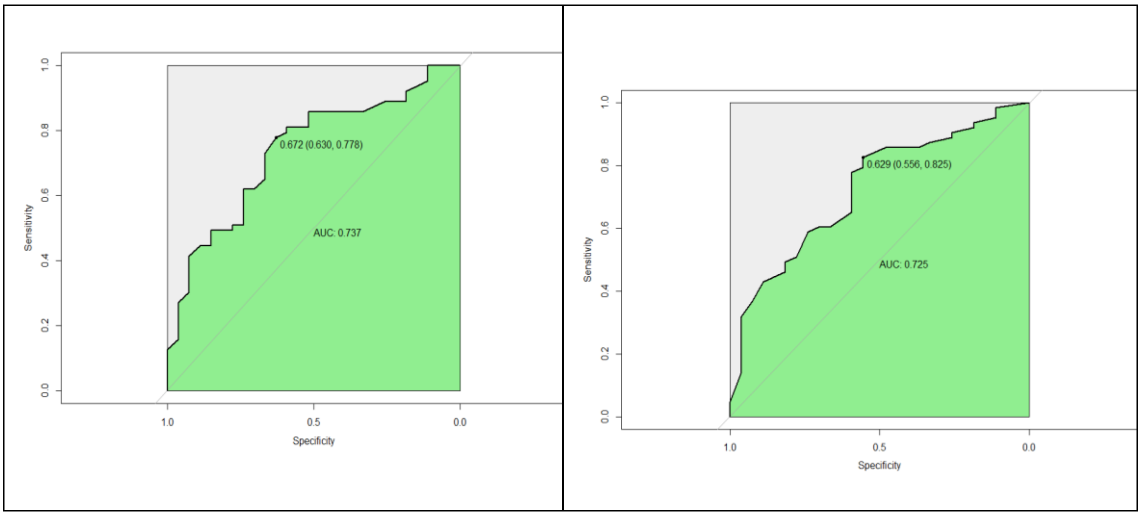

Figure S2: ROC curve of the adjusted models - model 1 and model 2.

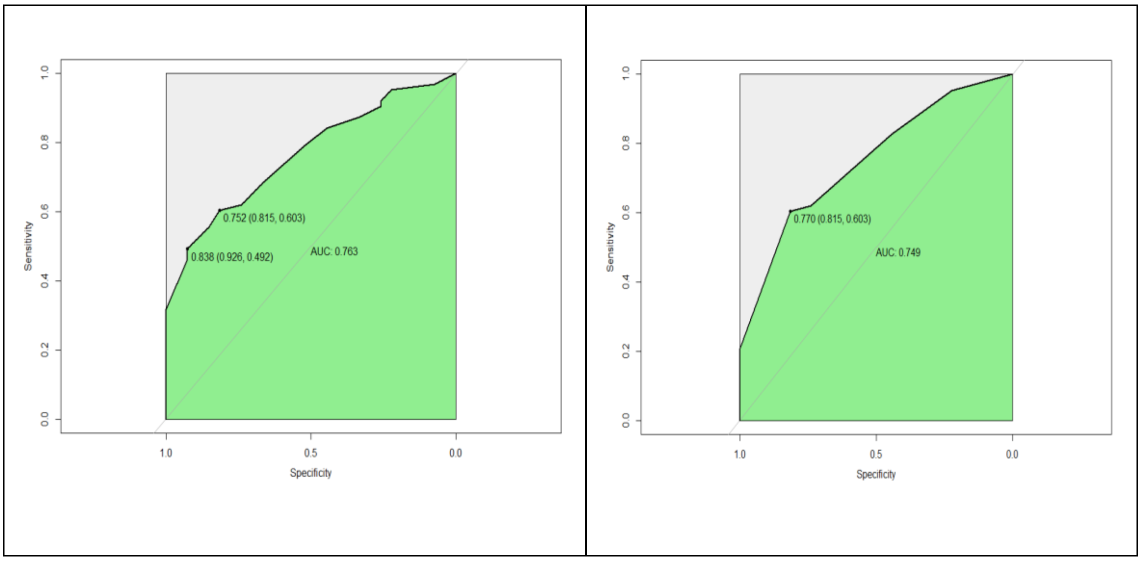

Supplement: Supplementary file 1 [file nutrients-15-03305-s001.zip › nutrients-2457707-supplementary.pdf]
